# P32 Relapsing polychondritis with anterior scleritis in young female

**DOI:** 10.1093/rap/rkab068.031

**Published:** 2021-10-19

**Authors:** Umair Arain, Abimbola Phillips, Ben Burton, Damodar Makkuni

**Affiliations:** James Paget, Great Yarmouth, United Kingdom

## Abstract

**Case report - Introduction:**

Relapsing polychondritis (RP) was first recognized as a clinical entity in 1923 by Jaksch-Wartenhorst (1923) and reported by him under the title "polychondropathia". The term "relapsing polychondritis" was first used by Pearson, Kline, and Newcomer (1960). Because the ocular findings can be the initial findings of RP, ophthalmologists should know the major ocular findings of this disease. Isaak et al reported that the most common ocular finding is episcleritis (39%) and the second is scleritis (14%). Other signs are iritis (9%), retinopathy (9%), muscle paresis (5%), and optic neuritis (5%).

**Case report - Case description:**

A 45-year-old female with known rheumatoid arthritis referred by rheumatology in eye clinic due to blurred vision and dry eye. The patient was on hydroxychloroquine and sulfasalazine. No retinal toxicity was found on examination, OCT and Visual Fields. The vision was 6/6 both eyes. Follow-up was in 12 months. She presented 6 months later in casualty with severe pain in her right eye. Examination showed diffuse anterior scleritis with secondary conjunctival inflammation. Anterior chamber cells present. Posterior segment showed no inflammation. Left eye was unremarkable. She was started on Froben 100mg tds with omeprazole. She was seen after a week and condition was improving. She was asked to taper off the meds. Inflammation resolved with 6/5 vision in both eyes and the next appointment was made in a year to monitor for hydroxychloroquine toxicity. In November 2020 she was seen by ENT with inflammation of the right ear cartilage. The pictures showed that the pinna was spared and cartilage was only involved. There was nasal crusting and stuffy nose but without any respiratory symptoms. She was prescribed 50mgs of prednisolone and this helped with her inflammation. She was seen by rheumatology later on and hydroxychloroquine and sulfasalazine was stopped, and she was started on methotrexate 10mgs weekly and folic acid 5mg weekly. Pulmonary function test and echocardiogram was ordered. The case was discussed in MDT rheumatology and it was decided that if joint symptoms got worse than biologics could be started. Methotrexate increased to 15mg subcut. Echocardiogram was normal with satisfactory blood tests. Her next appointment is in October 2021.

**Case report - Discussion:**

Initially the patient was diagnosed with rheumatoid arthritis with ocular inflammation (anterior scleritis) and was given the standard treatment of steroids to which the patient responded as well. Later when she developed the ear inflammation which involved only the cartilage the diagnosis was revised by rheumatology and changed to RP. As this is a rare life-threatening disease management was switched to immunosuppressive therapy to which she is currently responding well.

**Case report - Key learning points:**

It is important to consider the possibility that a rheumatology patient may have more than one diagnosis or be open to the idea of revising the diagnosis as the clinical picture evolves over the time. Given the nature of the disease all the systemic features should be examined thoroughly as any one missed area can lead to delayed diagnosis.

